# Supplementary material for: Global Trends in Research on Biological Control Agents of Drosophila suzukii: A Systematic Review
Source: Insects. 2025 Jan 30;16(2):133. doi: 10.3390/insects16020133 (PMC11856837; doi:10.3390/insects16020133)
Supplement: Supplementary file 1 [file insects-16-00133-s001.zip › insects-3355079-File S1.pdf]

PRISMA 2020 Checklist

Section and Topic	Item #	Checklist item	Location where item is reported
**TITLE**			
Title	1	Identify the report as a systematic review, meta-analysis, or both.	Title
**ABSTRACT**			
Abstract	2	See the PRISMA 2020 for Abstracts checklist.	Abstract
**INTRODUCTION**			
Rationale	3	Describe the rationale for the review in the context of existing knowledge.	Page 2. Introduction: Biological control of spotted wing drosophila is crucial to mitigate the economic and environmental damage caused by this invasive pest through sustainable management, using natural enmies, such as parasitoids, predators, and entomopathogens.
Objectives	4	Provide an explicit statement of the objective(s) or question(s) the review addresses.	We aimed to investigate current status, limitations, and research opportunities on spotted wing drosophila biological control through a systematic review, highlightining success cases.
**METHODS**			
Eligibility criteria	5	Specify the inclusion and exclusion criteria for the review and how studies were grouped for the syntheses.	We consider any articles that include the spotted wing drosophila (SWD) and publications that used one or more biological control agents of SWD in a pest control context.
Information sources	6	Specify all databases, registers, websites, organisations, reference lists and other sources searched or consulted to identify studies. Specify the date when each source was last searched or consulted.	Figure 1.<br>On January 3, 2024, we conducted a search in the Scopus and Web of Science Core Collection databases.
Search strategy	7	Present the full search strategies for all databases, registers and websites, including any filters and limits used.	This study used a systematic search in Scopus and Web of Science databases with terms like “spotted wing drosophila” AND “biological control” OR “natural enem*”. Peer-reviewed articles published between 2012 and 2023 in Portuguese, Spanish, or English were included, while reviews, books, and conference proceedings were excluded. Of 583 initial records, 184 met the eligibility criteria
Selection process	8	Specify the methods used to decide whether a study met the inclusion criteria of the review, including how many reviewers screened each record and each report retrieved, whether they worked independently, and if applicable, details of automation tools used in the process.	To determine whether a study met the eligibility criteria for the review, inclusion criteria were defined as publications on spotted wing drosophila and articles about biological control agents in the context of pest management, written in English, Spanish, or Portuguese, peer-reviewed, and published in journals between 2012 and 2023. Exclusion criteria included reviews, books, book chapters, conference papers, scientific notes, editorials, and publications outside the specified timeframe.
Data collection process	9	Specify the methods used to collect data from reports, including how many reviewers collected data from each report, whether they worked independently, any processes for obtaining or confirming data from study investigators, and if applicable, details of automation tools used in the process.	Searches were performed using the terms: "spotted wing drosophila" AND "biological control" OR "natural enem*" OR "parasitoid" OR "predator" OR "bacteria" OR "fungi" OR "virus" OR "nematode".
Data items	10a	List and define all outcomes for which data were sought. Specify whether all results that were	Not applicable

Section and Topic	Item #	Checklist item	Location where item is reported
		compatible with each outcome domain in each study were sought (e.g. for all measures, time points, analyses), and if not, the methods used to decide which results to collect.	
	10b	List and define all other variables for which data were sought (e.g. participant and intervention characteristics, funding sources). Describe any assumptions made about any missing or unclear information.	Not applicable
Study risk of bias assessment	11	Specify the methods used to assess risk of bias in the included studies, including details of the tool(s) used, how many reviewers assessed each study and whether they worked independently, and if applicable, details of automation tools used in the process.	Not applicable
Effect measures	12	Specify for each outcome the effect measure(s) (e.g. risk ratio, mean difference) used in the synthesis or presentation of results.	Not applicable
Synthesis methods	13a	Describe the processes used to decide which studies were eligible for each synthesis (e.g. tabulating the study intervention characteristics and comparing against the planned groups for each synthesis (item #5)).	Not applicable
	13b	Describe any methods required to prepare the data for presentation or synthesis, such as handling of missing summary statistics, or data conversions.	We used two criteria to analyze publication on the success of biological control, pro-vided by the systematic review method, considering as effective control based on the rec-ommendation of BCAs according to the conclusions of the analyzed articles and percent-age efficiency provided by the article: (1) studies with field performance, and (2) studies meeting criterion 1 with evidence of spotted-wing drosophila control. Efficiency was clas-sified as: < 30% (limited control), 30-50% (potential control), and > 50% (successful con-trol). Data extracted from these records were subjected to descriptive analysis.
	13c	Describe any methods used to tabulate or visually display results of individual studies and syntheses.	We analyzed the success of biological control using two criteria: (1) field performance studies and (2) those showing evidence of spotted-wing drosophila control. Efficiency was classified as <30% (limited), 30-50% (potential), and >50% (successful). The extracted data underwent descriptive analysis.
	13d	Describe any methods used to synthesize results and provide a rationale for the choice(s). If meta-analysis was performed, describe the model(s), method(s) to identify the presence and extent of statistical heterogeneity, and software package(s) used.	Not applicable
	13e	Describe any methods used to explore possible causes of heterogeneity among study results (e.g. subgroup analysis, meta-regression).	Not applicable
	13f	Describe any sensitivity analyses conducted to assess robustness of the synthesized results.	Not applicable
Reporting bias assessment	14	Describe any methods used to assess risk of bias due to missing results in a synthesis (arising from reporting biases).	Not applicable
Certainty assessment	15	Describe any methods used to assess certainty (or confidence) in the body of evidence for an outcome.	Not applicable
**RESULTS**			
Study selection	16a	Describe the results of the search and selection process, from the number of records identified in	Figure 2

Section and Topic	Item #	Checklist item	Location where item is reported
		the search to the number of studies included in the review, ideally using a flow diagram.	
	16b	Cite studies that might appear to meet the inclusion criteria, but which were excluded, and explain why they were excluded.	We identified 583 publication records using the search string terms, and after applying the filters, 399 publications were deemed inappropriate for information extraction, resulting in 184 publications eligible for analysis.
Study characteristics	17	Cite each included study and present its characteristics.	Our strategy search resulted in 583 publications. We excluded 399 publications after applying filters and screened 184 titles, keywords and abstracts.
Risk of bias in studies	18	Present assessments of risk of bias for each included study.	Not applicable
Results of individual studies	19	For all outcomes, present, for each study: (a) summary statistics for each group (where appropriate) and (b) an effect estimate and its precision (e.g. confidence/credible interval), ideally using structured tables or plots.	Figure 2, Supplementary sheet
Results of syntheses	20a	For each synthesis, briefly summarise the characteristics and risk of bias among contributing studies.	Not applicable
	20b	Present results of all statistical syntheses conducted. If meta-analysis was done, present for each the summary estimate and its precision (e.g. confidence/credible interval) and measures of statistical heterogeneity. If comparing groups, describe the direction of the effect.	Table 1 and 2
	20c	Present results of all investigations of possible causes of heterogeneity among study results.	Table 1 and 2
	20d	Present results of all sensitivity analyses conducted to assess the robustness of the synthesized results.	Table 1 and 2
Reporting biases	21	Present assessments of risk of bias due to missing results (arising from reporting biases) for each synthesis assessed.	Not applicable
Certainty of evidence	22	Present assessments of certainty (or confidence) in the body of evidence for each outcome assessed.	Not applicable
**DISCUSSION**			
Discussion	23a	Provide a general interpretation of the results in the context of other evidence.	This systematic review highlights the current understanding of biological control agents against *Drosophila suzukii*, with a primary focus on parasitoids such as *Trichopria drosophilae* and *Pachycrepoideus vindemmiae*, which have proven effective, particularly under laboratory conditions. Despite the potential of entomopathogens and predators, their field application faces environmental and ecological challenges. Most research is conducted in Europe and North America, with growing interest in South America and Asia. The study emphasizes the need to expand field experiments and combine approaches to overcome practical barriers, promoting sustainable alternatives to chemical pesticides.
	23b	Discuss any limitations of the evidence included in the review.	The main limitations of the review include the predominance of studies conducted in controlled environments, making it difficult to apply the results in the field due to environmental

Section and Topic	Item #	Checklist item	Location where item is reported
			variables. There is also a lack of research on the integration of different control agents and their compatibility in integrated pest management. Furthermore, the geographical concentration in Europe and North America limits applicability in other regions with distinct ecosystems, such as South America and Asia. The absence of studies in complex agricultural systems also represents a practical barrier.
	23c	Discuss any limitations of the review processes used.	The review presents limitations, such as focusing only on articles published in Portuguese, Spanish, and English, potentially excluding relevant studies in other languages. Additionally, the predominance of laboratory studies limits the practical applicability of the results in field conditions. The absence of a quality analysis of the included studies may also compromise the robustness of the conclusions and the reliability of the evidence presented.
	23d	Discuss implications of the results for practice, policy, and future research.	The review highlights the need for field validation of biological control agents (BCAs), like Trichopria drosophilae and Ganaspis kimorum, to address challenges in natural environments and adapt them to regional conditions. It emphasizes reducing reliance on chemical pesticides through sustainable pest management, urging policymakers to support field trials and develop regulatory frameworks. Future research should focus on integrating BCAs, enhancing international collaboration, and expanding studies in underrepresented regions to improve the practical application of BCAs globally.
**OTHER INFORMATION**			
Registration and protocol	24a	Provide registration information for the review, including register name and registration number, or state that the review was not registered.	This review was not registered in any systematic review registry.
	24b	Indicate where the review protocol can be accessed, or state that a protocol was not prepared.	This review was not registered in any systematic review registry.
	24c	Describe and explain any amendments to information provided at registration or in the protocol.	This review was not registered in any systematic review registry.
Support	25	Describe sources of financial or non-financial support for the review, and the role of the funders or sponsors in the review.	This work has supported by the National Council of Technological and Scientific Development of Brazil (CNPq) for the Scholarship of Research Productivity, USDA CRIS 2072-22000-044-00D, and the USDA ARS Area-wide Program (led by National Program Leader S. Young).
Competing interests	26	Declare any competing interests of review authors.	Conflicts of interest<br>None.
Availability of data, code and other materials	27	Report which of the following are publicly available and where they can be found: template data collection forms; data extracted from included studies; data used for all analyses; analytic code; any other materials used in the review.	Supplementary sheet containing the complete data extracted in the review process.

PRISMA 2020 Checklist

From: Page MJ, McKenzie JE, Bossuyt PM, Boutron I, Hoffmann TC, Mulrow CD, et al. The PRISMA 2020 statement: an updated guideline for reporting systematic reviews. BMJ 2021;372:n71. doi: 10.1136/bmj.n71.
This work is licensed under CC BY 4.0. To view a copy of this license, visit https://creativecommons.org/licenses/by/4.0/
